# Supplementary material for: A framework for decolonising and diversifying biomedical sciences curricula: rediscovery, representation and readiness
Source: FEBS Open Bio. 2024 Aug 2;14(11):1762–78. doi: 10.1002/2211-5463.13867 (PMC11532977; doi:10.1002/2211-5463.13867)
Supplement: Supplementary file 1 — Table S1. Responses to the question ‘How familiar are you with the idea of decolonising the curriculum at university?’ Table S2. Responses to the question ‘How important do you think it is to decolonise the curriculum in the Biomedical Sciences at Bristol?’ Table S3. Responses to the question ‘To what extent does your curriculum reflect the contribution of diverse cultures to the development of Biomedical Sciences?’ Table S4. Responses to the question ‘To what extent does your curriculum address the needs of diverse populations across the world?’ Table S5. Responses to the question ‘To what extent does your curriculum celebrate the contributions of people of colour and other minoritised groups to the development of biomedical sciences?’ Table S6. Responses to the question ‘To what extent does your curriculum draw on examples relating to other cultures and people of colour to illustrate ideas/concepts?’ Table S7. Student responses to the question ‘Do you feel represented by the science and scientists you learn about in the course?’ Table S8. Responses to the question ‘Are you aware of any activities currently taking place to try to decolonise the curriculum at [different levels]?’ Table S9. Student responses to the question ‘Are you aware of any activities currently taking place to try to decolonise the curriculum at [different levels]?’ Table S10. Staff responses to the question ‘Are you aware of any activities currently taking place to try to decolonise the curriculum at [different levels]?’ Table S11. Responses to the question ‘In your experience, do you feel that the Biomedical Sciences undergraduate curriculum is inclusive with respect to the following groups?’ Table S12. Student responses to the question ‘In your experience, do you feel that the Biomedical Sciences undergraduate curriculum is inclusive with respect to the following groups?’ Table S13. Staff responses to the question ‘In your experience, do you feel that the Biomedical Sciences undergraduate cur [file FEB4-14-1762-s001.pdf]

Supplementary information

Supplementary Table 1. Responses to the question 'How familiar are you with the idea of decolonising the curriculum at university?' 1 – never heard of it; 2 – some awareness; 3 – moderately aware; 4 – very aware; 5 – actively engaged in it. P values are from Mann-Whitney U tests for two-way comparisons and Kruskal-Wallis tests when there are more than two groups.

**How familiar are you with the idea of decolonising the curriculum at university?**				
**Demographic**	**Group**	**Median**	**N**	**P**
Staff vs Students	Students	2	121	<0.001
	Staff	4	71	
Ethnicity (students)	Asian	2	24	0.712
	Black	2	7	
	Mixed	2	9	
	Not listed	2	5	
	White	2	71	
Gender (students)	Female	2	80	0.943
	Male	2	36	
Sexuality (students)	LGBTQIA+	2	45	0.197
	Heterosexual	2	64	
Disability (students)	Disability	3	19	0.032
	No disability	2	96	
Religion (students)	Religious	2	37	0.645
	Not religious	2	80	
Year of study (students)	1	2	43	<0.001
	2	3	38	
	3	3	31	
	4	1	9	

Supplementary Table 2. Responses to the question ‘How important do you think it is to decolonise the curriculum in the Biomedical Sciences at Bristol?’ 1 – not important; 5 – very important. P values are from Mann-Whitney U tests for two-way comparisons and Kruskal-Wallis tests when there are more than two groups.

How important do you think it is to decolonise the curriculum in the Biomedical Sciences at Bristol?				
**Demographic**	**Group**	**Median**	**N**	**P**
Staff vs Students	Students	4	121	0.640
	Staff	4	71	
Ethnicity (students)	Asian	4	24	0.68
	Black	5	7	
	Mixed	4	9	
	Not listed	5	5	
	White	5	71	
Gender (students)	Female	5	80	<0.001
	Male	4	36	
Sexuality (students)	LGBTQIA+	4	45	0.701
	Heterosexual	5	64	
Disability (students)	Disability	4	19	0.817
	No disability	5	96	
Religion (students)	Religious	4	37	0.268
	Not religious	5	80	
Year of study (students)	1	5	43	0.751
	2	4.5	38	
	3	4	31	
	4	4	9	

Supplementary Table 3. Responses to the question 'To what extent does your curriculum reflect the contribution of diverse cultures to the development of Biomedical Sciences?' 1 – not at all; 5 – completely. P values are from Mann-Whitney U tests for two-way comparisons and Kruskal-Wallis tests when there are more than two groups.

**To what extent does your curriculum reflect the contribution of diverse cultures to the development of Biomedical Sciences?**				
**Demographic**	**Group**	**Median**	**N**	**P**
Staff vs Students	Students	3	121	0.679
	Staff	2	68	
Ethnicity (students)	Asian	2.5	24	0.137
	Black	1	7	
	Mixed	2	9	
	Not listed	3	5	
	White	3	71	
Gender (students)	Female	3	80	0.987
	Male	3	36	
Sexuality (students)	LGBTQIA+	2	45	0.836
	Heterosexual	3	64	
Disability (students)	Disability	2	19	0.358
	No disability	3	96	
Religion (students)	Religious	3	37	0.156
	Not religious	2	80	
Year of study (students)	1	2	43	0.476
	2	3	38	
	3	3	31	
	4	3	9	

Supplementary Table 4. Responses to the question 'To what extent does your curriculum address the needs of diverse populations across the world?' 1 – not at all; 5 – completely. P values are from Mann-Whitney U tests for two-way comparisons and Kruskal-Wallis tests when there are more than two groups.

To what extent does your curriculum address the needs of diverse populations across the world?				
**Demographic**	**Group**	**Median**	**N**	**P**
Staff vs Students	Students	3	121	0.994
	Staff	3	68	
Ethnicity (students)	Asian	3	24	0.672
	Black	2	7	
	Mixed	2	9	
	Not listed	3	5	
	White	3	71	
Gender (students)	Female	3	80	0.064
	Male	2	36	
Sexuality (students)	LGBTQIA+	2	45	0.075
	Heterosexual	3	64	
Disability (students)	Disability	3	19	0.795
	No disability	3	96	
Religion (students)	Religious	3	37	0.358
	Not religious	3	80	
Year of study (students)	1	3	43	0.274
	2	3	38	
	3	2	31	
	4	3	9	

Supplementary Table 5. Responses to the question 'To what extent does your curriculum celebrate the contributions of people of colour and other minoritised groups to the development of biomedical sciences?' 1 – not at all; 5 – completely. P values are from Mann-Whitney U tests for two-way comparisons and Kruksal-Wallis tests when there are more than two groups.

**To what extent does your curriculum celebrate the contributions of people of colour and other minoritised groups to the development of biomedical sciences?**				
**Demographic**	**Group**	**Median**	**N**	**P**
Staff vs Students	Students	2	121	0.580
	Staff	2	68	
Ethnicity (students)	Asian	2	24	0.844
	Black	1	7	
	Mixed	2	9	
	Not listed	2	5	
	White	2	71	
Gender (students)	Female	2	80	0.884
	Male	2	36	
Sexuality (students)	LGBTQIA+	2	45	0.627
	Heterosexual	2	64	
Disability (students)	Disability	2	19	0.281
	No disability	2	96	
Religion (students)	Religious	2	37	0.280
	Not religious	2	80	
Year of study (students)	1	2	43	0.704
	2	2	38	
	3	2	31	
	4	2	9	

Supplementary Table 6. Responses to the question 'To what extent does your curriculum draw on examples relating to other cultures and people of colour to illustrate ideas/concepts?' 1 – not at all; 5 – completely. P values are from Mann-Whitney U tests for two-way comparisons and Kruskal-Wallis tests when there are more than two groups.

**To what extent does your curriculum draw on examples relating to other cultures and people of colour to illustrate ideas/concepts?**				
**Demographic**	**Group**	**Median**	**N**	**P**
Staff vs Students	Students	2	121	0.077
	Staff	2	66	
Ethnicity (students)	Asian	2	24	0.099
	Black	1	7	
	Mixed	2	9	
	Not listed	2	5	
	White	2	71	
Gender (students)	Female	2	80	0.740
	Male	2	36	
Sexuality (students)	LGBTQIA+	2	45	0.903
	Heterosexual	2	64	
Disability (students)	Disability	2	19	0.913
	No disability	2	96	
Religion (students)	Religious	2	37	0.382
	Not religious	2	80	
Year of study (students)	1	2	43	0.988
	2	2	38	
	3	2	31	
	4	2	9	

Supplementary Table 7. Student responses to the question 'Do you feel represented by the science and scientists you learn about in the course?' 1 – not at all; 5 – completely. P values are from Mann-Whitney U tests for two-way comparisons and Kruskal-Wallis tests when there are more than two groups.

**Do you feel represented by the science and scientists you learn about in the course?**				
**Demographic**	**Group**	**Median**	**N**	**P**
All Students	Students	3	121	-
Ethnicity (students)	Asian	2	24	<0.001
	Black	1	7	
	Mixed	2	9	
	Not listed	2	5	
	White	4	71	
Gender (students)	Female	3	80	0.156
	Male	3	36	
Sexuality (students)	LGBTQIA+	3	45	0.877
	Heterosexual	3	64	
Disability (students)	Disability	3	19	0.737
	No disability	3	96	
Religion (students)	Religious	2	37	0.002
	Not religious	3	80	
Year of study (students)	1	3	43	0.117
	2	3	38	
	3	3	31	
	4	4	9	

Supplementary Table 8. Responses to the question 'Are you aware of any activities currently taking place to try to decolonise the curriculum at [different levels]?' Comparison of student and staff responses with Pearson's chi square analysis.

**Are you aware of any activities currently taking place to try to decolonise the curriculum at UK Universities?**				
	**No**	**Yes**	**Total**	**Pearson's chi square**
Staff	28	36	64	0.216
Students	63	54	117	
Total	91	90	181	
**Are you aware of any activities currently taking place to try to decolonise the curriculum at the University of Bristol?**				
	**No**	**Yes**	**Total**	**Pearson's chi square**
Staff	14	54	68	0.002
Students	52	68	120	
Total	66	122	188	
**Are you aware of any activities currently taking place to try to decolonise the curriculum in the Faculty of Life Sciences?**				
	**No**	**Yes**	**Total**	**Pearson's chi square**
Staff	12	54	66	<0.001
Students	75	42	117	
Total	87	96	183	
**Are you aware of any activities currently taking place to try to decolonise the curriculum in your School?**				
	**No**	**Yes**	**Total**	**Pearson's chi square**
Staff	11	60	71	<0.001
Students	77	39	116	
Total	88	99	187	
**Are you aware of any activities currently taking place to try to decolonise the curriculum in your units?**				
	**No**	**Yes**	**Total**	**Pearson's chi square**
Staff	23	41	64	<0.001
Students	90	24	114	
Total	113	65	178	

Supplementary Table 9. Student responses to the question 'Are you aware of any activities currently taking place to try to decolonise the curriculum at [different levels]?' Comparison of students' responses to different categories with Pearson's chi square analysis and post-hoc tests. Bonferroni adjusted alpha value = 0.005 for 10 comparisons.

**Are you aware of any activities currently taking place to try to decolonise the curriculum at…?**					
	No	Yes	Total	P value	Post-hoc P value
**UK universities**	63	54	117	<0.001	0.0719
**UoB**	52	68	120		<0.001
**Faculty of Life Sciences**	75	42	117		0.484
**School**	77	39	116		0.194
**Units**	90	24	114		<0.001

Supplementary Table 10. Staff responses to the question 'Are you aware of any activities currently taking place to try to decolonise the curriculum at [different levels]?' Comparison of staff responses to different categories with Pearson's chi square analysis and post-hoc tests. Bonferroni adjusted alpha value = 0.005 for 10 comparisons.

**Are you aware of any activities currently taking place to try to decolonise the curriculum at…?**					
	No	Yes	Total	P value	Post-hoc P value
**UK universities**	28	36	64	<0.001	<0.001
**UoB**	14	54	68		0.230
**Faculty of Life Sciences**	11	60	71		0.0164
**School**	12	54	66		0.0891
**Units**	23	41	64		0.0574

Supplementary Table 11. Responses to the question 'In your experience, do you feel that the Biomedical Sciences undergraduate curriculum is inclusive with respect to the following groups?' Comparison of student and staff responses with Pearson's chi square analysis.

**In your experience, do you feel that the Biomedical Sciences undergraduate curriculum is inclusive with respect to the following groups?**				
		**People of colour**		
	**No**	**Yes**	**Total**	**Pearson's chi square**
Staff	39	22	61	0.057
Students	56	60	116	
Total	95	82	177	
		**People from the Global South**		
	**No**	**Yes**	**Total**	**Pearson's chi square**
Staff	44	18	62	0.038
Students	63	52	115	
Total	107	70	177	
		**Women**		
	**No**	**Yes**	**Total**	**Pearson's chi square**
Staff	24	36	60	0.003
Students	21	94	115	
Total	45	130	175	
		**LGBTQIA+ community**		
	**No**	**Yes**	**Total**	**Pearson's chi square**
Staff	37	24	61	0.057
Students	51	64	115	
Total	88	88	176	
		**People with a disability**		
	**No**	**Yes**	**Total**	**Pearson's chi square**
Staff	45	16	61	0.096
Students	68	45	113	
Total	113	61	174	
		**People of certain religions**		
	**No**	**Yes**	**Total**	**Pearson's chi square**
Staff	36	23	59	0.197
Students	55	55	110	
Total	91	78	169	

Supplementary Table 12. Student responses to the question 'In your experience, do you feel that the Biomedical Sciences undergraduate curriculum is inclusive with respect to the following groups?' Comparison of students' responses to different categories with Pearson's chi square analysis and post-hoc tests. Bonferroni adjusted alpha value = 0.0042 for 12 comparisons.

	No	Yes	Total	P value	Post-hoc P value
**People with a disability**	68	45	113		<0.001
**People from the Global South**	63	52	115		0.0357
**LGBTQIA+ community**	51	64	115	<0.001	0.689
**People of colour**	56	60	116		0.549
**People with certain religious beliefs**	55	55	110		0.368
**Women**	21	94	115		<0.001

Supplementary Table 13. Staff responses to the question 'In your experience, do you feel that the Biomedical Sciences undergraduate curriculum is inclusive with respect to the following groups?' Comparison of staff responses to different categories with Pearson's chi square analysis and post-hoc tests. Bonferroni adjusted alpha value = 0.0042 for 12 comparisons.

	No	Yes	Total	P value	Post-hoc P value
**People with a disability**	45	45	113		<0.001
**People from the Global South**	44	52	115		0.0357
**LGBTQIA+ community**	37	64	115	0.003	0.689
**People of colour**	39	60	116		0.549
**People with certain religious beliefs**	36	55	110		0.368
**Women**	24	36	115		<0.001

Staff survey questions

Part 1: demographic information

Q1. Which is your home School?
Biochemistry
CMM
PPN

Q2. What is your academic career pathway[1]?
Pathway 1
Pathway 2
Pathway 3
Prefer not to say

Q3. Do you consider yourself to be a member of a minoritised group?
Yes
No
Prefer not to say

Q3a. If yes, please tell us which minoritised group or groups you belong to, if you feel comfortable doing so.

Part 2: decolonising the curriculum

Q4. How familiar are you with the idea of decolonising the curriculum at university?
Never heard of it
Some awareness
Moderately aware
Very aware
Actively engaged in it

Decolonisation is broadly about confronting how colonialism, Eurocentrism, and racism have shaped our world. Decolonisation of the curriculum recognises these issues in the science we teach and aims to include a range of perspectives of people who have been overlooked, so as to present a truer picture of our discipline so that it can be more responsive to the needs of all.

Q5. Are you aware of any activities currently taking place to try to decolonise the curriculum at the following levels?

	Yes	No
UK universities		
University of Bristol		
Faculty of Life Sciences		
My School		
My units		

Q6. How important do you think it is to decolonise the curriculum in the Biomedical Sciences at the University of Bristol?
Not important 1 2 3 4 5 very important

Q7. In your experience, to what extent does the curriculum within the Biomedical Schools (Biochemistry, CMM, or PPN) reflect the contribution of diverse cultures to the development of biomedical sciences?
Not at all 1 2 3 4 5 Completely

Q8. In your experience, to what extent does the curriculum within the Biomedical Schools address the needs of diverse populations around the world?
Not at all 1 2 3 4 5 Completely

[1] There are three academic career pathways at the University of Bristol: Pathway 1 (research and teaching focused); Pathway 2 (research focused) and Pathway 3 (teaching and scholarship focused)

Q9. In your experience, to what extent does the curriculum within the Biomedical Schools celebrate the contributions of people of colour and other minoritised groups to the development of biomedical sciences?
Not at all 1 2 3 4 5 Completely

Q10. In your experience, to what extent does the curriculum within the Biomedical Schools draw on examples relating to other cultures and people of colour to illustrate ideas/ concepts?
Not at all 1 2 3 4 5 Completely

Q11. What three things would you expect to see in a decolonised curriculum? [Max 280 characters]

Part 3 – Equality, Diversity and Inclusion in the Biomedical Sciences curriculum
Q12 Do you feel all students are represented by the science and scientists that students learn about in the course?
Not at all 1 2 3 4 5 Completely

Q13. In your experience, do you feel that the Biomedical Sciences curriculum (School of Biochemistry, CMM or PPN) is inclusive with respect to the following groups?

	Yes	No
People of colour		
People from the Global South		
Women		
LGBTQIA community		
People with a disability		
People of certain religions		
Not listed (please describe) ______________		

Q14. Do you feel comfortable discussing issues of inclusivity, bias or inequality with students in the courses on which you teach?
- Very comfortable
- Somewhat comfortable
- Neither comfortable nor uncomfortable
- Somewhat uncomfortable
- Very uncomfortable
- Not aware of any

Q15. If you have experienced any non-inclusive practice (such as bias or inequality) at the university, at which level of the organisation was it? Select all that apply:
- Not experienced any non-inclusive practice
- Taught units
- Wider curriculum
- Personal tutoring
- University infrastructure or senior management
- Other_____________

Q16. If you are made aware of any non-inclusive practice (such as bias or inequality) in the courses in your school, would you feel comfortable raising it?
- Very comfortable
- Somewhat comfortable
- Neither comfortable nor uncomfortable
- Somewhat uncomfortable
- Very uncomfortable

Q17. To whom would you feel it is best to raise these issues if you had them? *Select all answers which apply.*

- Individual lecturers
- Unit Director
- School Education Director
- Faculty Education Director
- Head of School
- School Equality, Diversity and Inclusion Committee
- Other_____________

Part 4 - Final comments
Q18. Do you have any other comments relating to decolonising and diversifying the curriculum?

Q19. Would you be interested in working with the School/Faculty on decolonising and diversifying the curriculum in the future?
Yes
No

Thanks for completing the survey!

Student survey questions

Part 1: demographic information

Q1. Which School is your programme associated with?
Biochemistry
Biomedical Sciences
CMM
PPN

Q2. What year of study are you in?
Foundation year
Year 1
Year 2
Year 3
Year 4

Q3. What is your ethnicity?
Asian/Asian British
Black/African/Caribbean/Black British
White
Mixed/multiple ethnic groups – white and Asian
Mixed/multiple ethnic groups – white and Black
Mixed/multiple ethnic groups – other mixed/multiple ethnic groups, please describe
Not listed – please describe
Prefer not to say

Q4. What is your gender?
Female
Male
Non-binary
Not listed (please describe)
Prefer not to say

Q4a. Is this the gender you were assigned at birth?
Yes
No
Prefer not to say

Q5. What is your sexuality?
Bisexual
Gay/lesbian
Straight/heterosexual
Not listed (please describe)
Prefer not to say

Q6. Do you consider yourself to have a disability?
Yes
No
Prefer not to say

Q7. What is your religion, if you have one?
No religion
Buddhist
Christian
Hindu
Jewish
Muslim

Sikh
Not listed (please describe)
Prefer not to say

Part 2: decolonising the curriculum
Q8. How familiar are you with the idea of decolonising the curriculum at university?
Never heard of it
Some awareness
Moderately aware
Very aware
Actively engaged in it

Decolonisation is broadly about confronting how colonialism, Eurocentrism, and racism have shaped our world. Decolonisation of the curriculum recognises these issues in the science we teach and aims to include a range of perspectives of people who have been overlooked, so as to present a truer picture of our discipline so that it can be more responsive to the needs of all.

Q9. Are you aware of any activities currently taking place to try to decolonise the curriculum at the following levels?

	Yes	No
UK universities		
University of Bristol		
Faculty of Life Sciences		
My School		
My units		

Q10. How important do you think it is to decolonise the curriculum in the Biomedical Sciences at the University of Bristol?
Not important 1 2 3 4 5 very important

Q11. To what extent does your curriculum reflect the contribution of diverse cultures to the development of biomedical sciences?
Not at all 1 2 3 4 5 Completely

Q12. To what extent does your curriculum address the needs of diverse populations around the world?
Not at all 1 2 3 4 5 Completely

Q13. To what extent does your curriculum celebrate the contributions of people of colour and other minoritised groups to the development of biomedical sciences?
Not at all 1 2 3 4 5 Completely

Q14. To what extent does your curriculum draw on examples relating to other cultures and people of colour to illustrate ideas/ concepts?
Not at all 1 2 3 4 5 Completely

Q15. What three things would you expect to see in a decolonised curriculum? [Max 280 characters]

Part 3 – Equality, Diversity and Inclusion in the Biomedical Sciences curriculum
Q16 Do you feel represented by the science and scientists you learn about in the course?
Not at all 1 2 3 4 5 Completely

Q17. Do you feel that your curriculum is inclusive with respect to the following groups?

	Yes	No
People of colour		
People from the Global South		
Women		

LGBTQIA community		
Disabled people		
People of certain religions		
Other ________________		

Q18. Do you feel comfortable raising issues about inclusivity, bias or inequality in your course material?

- Very comfortable
- Somewhat comfortable
- Neither comfortable nor uncomfortable
- Somewhat uncomfortable
- Very uncomfortable
- Not aware of any

Q19. To whom would you feel it is best to report these issues if you had them?

- Unit Director
- Individual lecturers
- Personal tutor
- Senior tutor
- Students Union
- Student Reps/SSLC
- University online reporting tool
- Other_____________

Q20. If you have experienced any non-inclusive practice (such as bias or inequality) at university, at which level of the organisation was it? Select all that apply:

- Not experienced any non-inclusive practice
- Taught units
- Wider curriculum
- Personal tutoring
- Student societies such as Helix, The Cell, Biomedical Sciences Society, Neuroscience Society etc
- University infrastructure or senior management
- Other_____________

Q21 Are there any specific units or topics in which you have experienced non-inclusive practice? Please explain your answer. Please do not name any individuals. If you wish to raise any concerns about unacceptable behaviour, please do so here Report + Support - University of Bristol

Q22. Have you come across any examples of good practice where inequalities have been highlighted and discussed within your course? Please explain them.

Q23 Are there any ways a discussion about equality, diversity and inclusion in science can be encouraged within your course?

Part 4 - Final comments

Q24. Do you have any other comments relating to decolonising and diversifying the curriculum?

Q25. Would you be interested in working with the School/Faculty on decolonising and diversifying the curriculum in the future?

Yes

No

Thanks for completing the survey!
